# Machine learning to extract physiological parameters from multispectral diffuse reflectance spectroscopy

**DOI:** 10.1117/1.JBO.26.5.052912

**Published:** 2021-03-17

**Authors:** Mayna H. Nguyen, Yao Zhang, Frank Wang, Jose De La Garza Evia Linan, Mia K. Markey, James W. Tunnell

**Affiliations:** a The University of Texas at Austin, Department of Biomedical Engineering, Austin, Texas, United States; b The University of Texas MD Anderson Cancer Center, Houston, Texas, United States

**Keywords:** diffuse reflectance spectroscopy, optical properties, Monte Carlo methods, machine learning

## Abstract

**Significance:** Physiological parameters extracted from diffuse reflectance spectroscopy (DRS) provide clinicians quantitative information about tissue that helps aid in diagnosis. There is a great need for an accurate and cost-effective method for extracting parameters from DRS measurements.

**Aim:** The aim is to explore the accuracy and speed of physiological parameter extraction using machine learning models compared to that of the widely used Monte Carlo lookup table (MCLUT) inverse model.

**Approach:** Diffuse reflectance spectra were simulated using a light transport model based on Monte Carlo simulations and weighted to six wavelengths. Deep learning (DL), random forest (RF), gradient boosting machine (GBM), and generalized linear model (GLM) machine learning models were built using a training set of 10,000 spectra from the simulated data. The MCLUT and machine learning models were used to predict physiological parameters from a separate test set of 30,000 simulated spectra. Mean absolute errors were calculated to evaluate the accuracy and compare it among MCLUT and machine learning models. In addition, the computational time to predict parameters from the test set was recorded to compare the speed among MCLUT and machine learning models.

**Results:** The DL, RF, GBM, and GLM models all had significantly lower errors than the MCLUT inverse method for six wavelengths. The DL model proved to have the lowest errors, with all absolute percent errors under 10%. The DL model had much faster runtimes than the MCLUT.

**Conclusions:** Machine learning is promising for extracting physiological parameters from six-wavelength DRS data, with both lower errors and a faster runtime than the widely used MCLUT model.

## 1 Introduction

Diffuse reflectance spectroscopy (DRS) is an optical technology that uses light to non-invasively measure optical properties of biological tissue and has applications in the diagnosis of several cancers such as breast,^1^ colorectal,^2^ cervical,^3^^,^^4^ oral,^5^^,^^6^ lung,^7^ and skin.^8^^–^^10^ DRS instruments typically use an optical fiber probe to emit light onto tissue, where the light is scattered and absorbed. The light is then collected back into the fiber and is sent into a spectrometer to return reflectance values to a computer. A full spectrum of wavelengths is typically used to capture sufficient data for an accurate analysis of tissue composition. Example full-spectrum devices include Dermasensor (Dermasensor, Miami, Florida), a handheld device for performing reflectance measurements of skin, and Zenascope IM1 (Zenalux, Durham, North Carolina), a DRS system that measures biological endpoints of tissue for various diagnostic applications. These full-spectrum systems can be expensive due to the high cost of spectrometers, which can drive the cost of even the cheaper DRS systems to be around $2600 to $3800.^11^ Thus, costs can potentially be reduced by utilizing a cheaper spectrometer that has a limited number of wavelengths.

To help clinicians understand the characteristics of tissue, optical properties and physiological parameters can be extracted from the DRS data. Previous studies have developed inverse models using lookup tables that are built from experimental data^12^ to extract such parameters, but recently, lookup tables that are built from Monte Carlo simulations^8^^,^^13^^,^^14^ have been shown to be more efficient for extracting parameters. However, these Monte Carlo lookup table (MCLUT)-based models traditionally use a full spectrum of wavelengths, and it is unknown if using fewer wavelengths affects the accuracy. Additionally, a limitation of the MCLUT-based inverse model is that it uses a time-consuming iterative process to fit the DRS measurement data to determine optical properties, which is not ideal for processing large sample sizes or for use in the clinic.

Previous studies utilized machine learning to obtain optical properties from diffuse reflectance. Barman et al.^15^ used least-squares support vector machines to determine the absorption and reduced scattering coefficients from diffuse reflectance values more accurately and faster than the MCLUT-based inverse model. Tsui et al.^16^ developed forward artificial neural network models to estimate optical and physiological information from spatially resolved diffuse reflectance spectra of multi-layered tissue. Panigrahi et al.^17^ used a random forest (RF) model to estimate optical properties from diffuse reflectance in the spatial frequency domain. These studies show that machine learning models have the potential to accurately predict parameters for complex problems, and therefore could be useful for reducing the dependence on a large number of wavelengths.

The AS7262 6-Channel Visible Spectral ID Device (ams, Premstätten, Austria) is a multi-spectral sensor chip that captures six specific wavelengths. In this study, we use this chip as a reference and explore the value of using machine learning to extract physiological parameters from the six wavelengths specified by the chip. We show that while the MCLUT inverse model is not sufficient to extract physiological parameters from six wavelengths of diffuse reflectance spectra, machine learning models provide an alternative method to accurately and quickly extract parameters.

## 2 Materials and Methods

### 2.1 Monte Carlo Lookup Table

The Monte Carlo lookup table was built by simulating photon transport in tissue to map reflectance values to pairs of absorption and reduced scattering coefficients. Each simulation used 1,000,000 photons with combined pairs of 40 evenly spaced absorption coefficients ($0\text{–}50\;{\mathrm{cm}}^{-1}$, $\text{step size}=2.56\;{\mathrm{cm}}^{-1}$) and 40 evenly spaced reduced scattering coefficients ($0\text{–}50\;{\mathrm{cm}}^{-1}$, $\text{step size}=2.56\;{\mathrm{cm}}^{-1}$) to result in a 40×40 MCLUT. More details can be found in previous literature.^8^^,^^13^ Briefly, light propagation in tissue is simulated by using repeated random sampling of probability distributions based on scattering angles and step sizes to describe photon movement. The result of many simulations estimates the reflectance intensity as the number of photons that are measured by the detector over the number of photons launched.

### 2.2 Diffuse Reflectance Spectra Simulation and Data Generation

Diffuse reflectance spectra were simulated in MATLAB (R2019b; MathWorks Inc., Natick, MA) for 100 wavelengths from 410 to 650 nm. The MCLUT-based forward model used one of 10 linearly spaced values of each physiological parameter within the ranges found in Table 1 to simulate a 100-wavelength reflectance spectrum, for a total of 100,000 spectra. The values were linearly spaced to get a variation of simulated spectra in all parameters and to avoid potential overlap in the test and training sets. 10 values were chosen for each parameter to maintain a balance between the sample size (100,000 spectra) and the computation time of the MCLUT models (a few hours for simulating the spectra). The physiological parameters used were blood volume fraction (BVF), reduced scattering coefficient (${\mu_{s}}^{\prime }$), reduced scattering exponent (B), melanin concentration (Mel), and oxygen saturation ($O_{2}$). The parameters used and the corresponding ranges were selected based on physiological ranges for skin tissue.^8^^,^^12^ The equations used for the MCLUT forward model are reported in the previous physiological model.^8^ Briefly, using ${\mu_{s}}^{\prime }$ at 630 nm and B, the ${\mu_{s}}^{\prime }$ at any wavelength can be calculated. BVF, Mel, and $O_{2}$ contribute to the absorption coefficient calculation. The 100-wavelength spectra were simulated with Gaussian random noise to mimic realistic DRS data. Other types of noise were also simulated and tested but did not significantly change the results (see Table S1 in the Supplementary Material). Therefore, the results from Gaussian noise spectra are reported here.

**Table 1:** Physiological parameter value ranges.

Physiological parameter	Value ranges
BVF: blood volume fraction	1–5%
${\mu_{s}}^{\prime }$: reduced scattering coefficient	$1030\;{\mathrm{cm}}^{-1}$
B: reduced scattering exponent	1.3–2.5
Mel: melanin concentration	02 mg/ml
$O_{2}$: oxygen saturation	60–100%

**Fig. 1:**
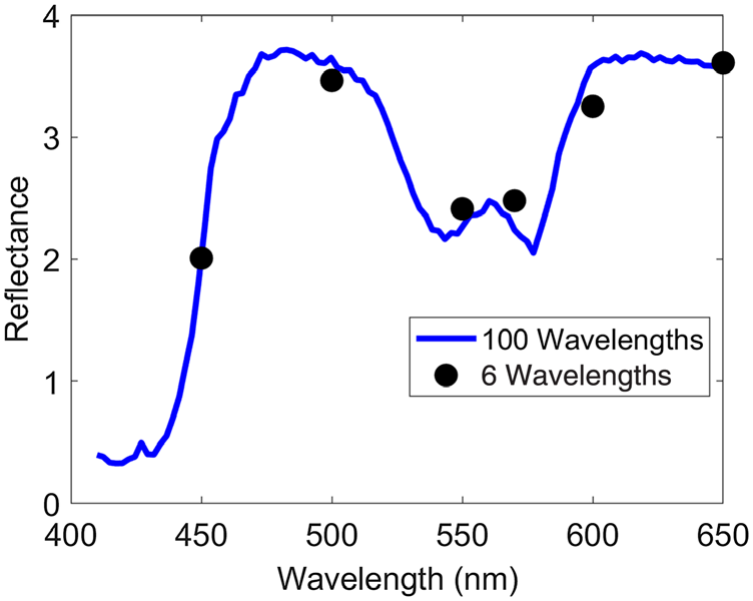
Simulated diffuse reflectance spectra for 100 wavelengths and 6 wavelengths using physiological parameter values BVF=1%, ${\mu_{s}}^{\prime }=10\;{\mathrm{cm}}^{-1}$, B=1.3, Mel=0  mg/ml, and $O_{2}=60\%$. The resultant weighted average 6-wavelength spectra capture the general shape but does not provide the full information that the 100-wavelength spectra give.

The diffuse reflectance spectra were then calculated for the following six wavelengths as used by the AS7262 6-Channel device: 450, 500, 550, 570, 600, and 650 nm. Because the bandwidth of each channel in the AS7262 is broad, the down-sampled spectrum was computed using a weighted average according to the device responsivity (see Fig. S1 in the Supplementary Material). Normal distributions with mean of 40 and standard deviation of 2.35 were used to calculate the weighted average reflectance values for six wavelengths. One representative example of simulated spectra with 100 wavelengths and the corresponding weighted average six-wavelength spectra is shown in Fig. 1.

To minimize overfitting the machine learning models, cross validation was done by splitting the dataset into a training set and a test set. The six-wavelength dataset was randomized into a training set of 70,000 spectra and a test set of 30,000 spectra. The training set was later reduced to 10,000 spectra, which allowed for both sufficient training time (roughly 5 to 10 s per parameter per model) and accuracy. The same 10,000 spectra were used for training all machine learning models and the same 30,000 spectra were used to test the MCLUT model and all machine learning models.

### 2.3 MCLUT Inverse Model

The MCLUT inverse model^8^^,^^13^ uses nonlinear optimization to minimize the error between the measured reflectance and the simulated reflectance in the MCLUT. The *fmincon* MATLAB function with the interior-point algorithm was used to minimize the error and fit the spectra. The final extracted physiological parameters are the result of the error reaching the minimum on the test dataset.

### 2.4 Machine Learning Models

Four different machine learning models were trained and tested for parameter extraction. The h2o package from H2O.ai, an open-source machine learning package, was used in R (RStudio 3.5.1) to create the models because of H2O’s ease of use, compatibility with multiple languages, ability to scale to large data, and library of widely used machine learning algorithms.^18^ This allows for the development of several models to assess the robustness of machine learning for a specific application. Additionally, H2O has several built-in parameters that were used to limit overfitting the models to ensure accurate results such as the low default number of trees, limiting the number of epochs, and using the right amount of regularization. Separate models were built for each of the five physiological parameters using deep learning (DL), RF, gradient boosting machine (GBM), and generalized linear models (GLM), respectively. 10,000 spectra of the training dataset were used to train the models. Each model was trained with H2O’s default settings and was tested for parameter extraction on the test set. A general overview of these machine learning algorithms is described below, with more information in the H2O documentation and resources.

H2O’s DL involves a using feedforward artificial neural network with multiple hidden layers of neurons (default = two hidden layers of 200 neurons each) to learn the features of a dataset. Each neuron in a layer produces a weighted combination of its inputs. During learning, the weights are adjusted with the goal of minimizing the error on the training data. H2O’s DL model has the option of choosing between tanh, rectifier, and maxout activation functions. The default activation function is rectifier. The default epoch value is 10, which is the number of times to iterate the dataset.^19^ RF models develop a forest of multiple decision trees based on random subsets of data. Each tree has some error and when combined, the large number of uncorrelated trees (default = 50 trees) work together to outperform individual trees and lower the variance. The average prediction over all the trees is the final regression prediction. The maximum tree depth default is 20 trees and the default number of bins for the histogram to build then split is 20.^20^ H2O’s GBM model builds regression trees (default = 50 trees) on the dataset features in stages and adds a new model that is trained based on the error of the previous set of models. With each new model, the approximation of the response variable is more refined. The maximum tree depth default is five trees and the default number of bins for the histogram to build then split is 20.^18^^,^^20^ GLMs are extensions of traditional linear regressions and can be used for response variables that follow distributions other than just the normal distribution. H2O’s GLM includes distributions such as binomial, Gaussian, Poisson, and more and fits the model based on the maximum likelihood estimation via iteratively reweighed least squares.^20^ The default Gaussian distribution, which fits a traditional linear regression model, was used because the response variable error distributions were unknown.

H2O allows for a simplified way to build and compare machine learning models and was used initially for this purpose. However, it is not a fair comparison for a time analysis since the H2O models are in R while the MCLUT is in MATLAB. For a more comparable time analysis, a DL model was built in MATLAB for each parameter after it was determined that the H2O DL model performed the best. MATLAB’s Neural Net Fitting tool in the Deep Learning toolbox was used to build a model that resembled the model that was built in H2O as close as possible. MATLAB has different training algorithms from H2O and the Levenberg–Marquardt was used because it stops the training when the mean square error stops improving, similar to how DL in H2O works. One hidden layer of 200 neurons was specified and training was stopped after 10 epochs.

### 2.5 Evaluation Metrics

The mean absolute error for each parameter is calculated as

Mean Absolute Error=mean(|Predict−Truth|),

and the mean absolute percent error is calculated as

$$Mean\;Absolute\;Percent\;Error=mean(\frac{\left|Predict-Truth\right|}{Truth}*100).$$

Bar plots of the mean absolute error created in R are presented because the absolute percent errors created variability in the errors when dividing by small parameter values. Mean absolute percent error is reported to compare to errors reported in previous studies. For a detailed look at the distribution of the absolute percent error for each parameter value, refer to Fig. S2 in the Supplementary Material. The R package afex^21^ was used to do a linear mixed effects analysis of the extracted parameters from each model compared to the truth values of the parameters.

The average runtime was calculated for each trained model for 1, 10, 50, and 100 spectra using 50 trials of randomly selected spectra from the test dataset. The parameter extraction was run on a 2-core Intel Core i5-5200U CPU (2.20 GHz) with 8 GB RAM and a 64-bit Windows 10 Operating System using R for the H2O machine learning models and MATLAB for the MCLUT model and the DL model built in MATLAB.

## 3 Results

**Fig. 2:**
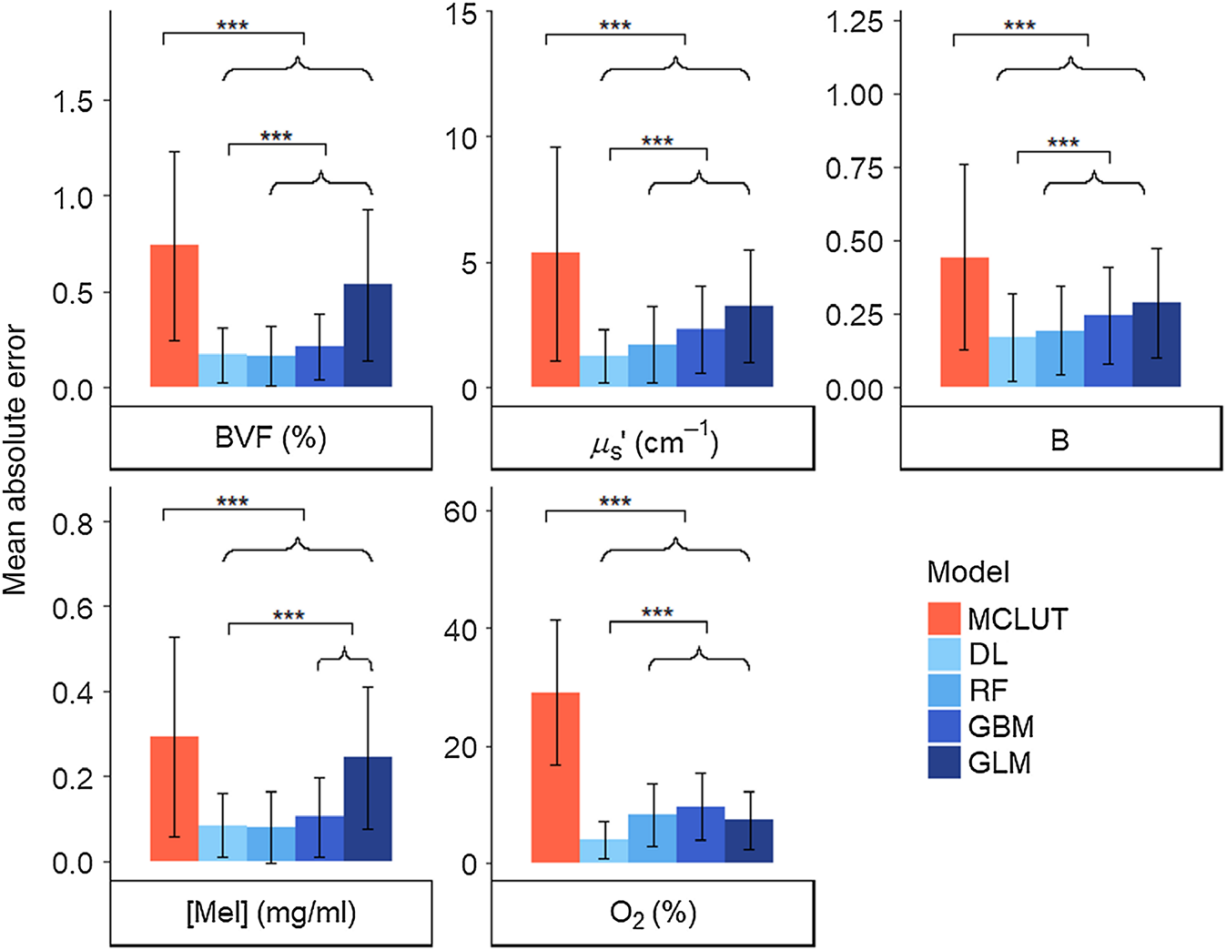
Mean absolute error for extracted physiological parameters for all models: MCLUT inverse model, DL, RF, GBM, and GLM. Error bars represent standard deviation. *** means p<001. The MCLUT model has significantly higher errors than all machine learning models. The DL model has the lowest errors out of all models, with the exception of the Mel parameter.

The accuracy of each model was calculated first to compare how the machine learning models perform relative to the MCLUT inverse model. The mean absolute errors for each physiological parameter using each model are shown in Fig. 2. From the mixed model, all machine learning models were shown to have significantly lower errors than MCLUT for all parameters. The DL model errors were also significantly different than the RF, GBM, and GLM models for all parameters except Mel, where the error was not significantly different from RF. Since the DL model performs the best apart from the Mel parameter, this model will be used as the focus of the remainder of the results. The mean absolute percent errors for the DL model were 5.15% for BVF, 6.88% for ${\mu_{s}}^{\prime }$, 9.81% for B, 9.91% for Mel, and 4.61% for $O_{2}$. Crosstalk between the fit parameters was examined by determining the Pearson correlation coefficient between a parameter truth value and the other resulting extracted parameters. There was no significant correlation between any of the parameters (r<±0.3).

**Fig. 3:**
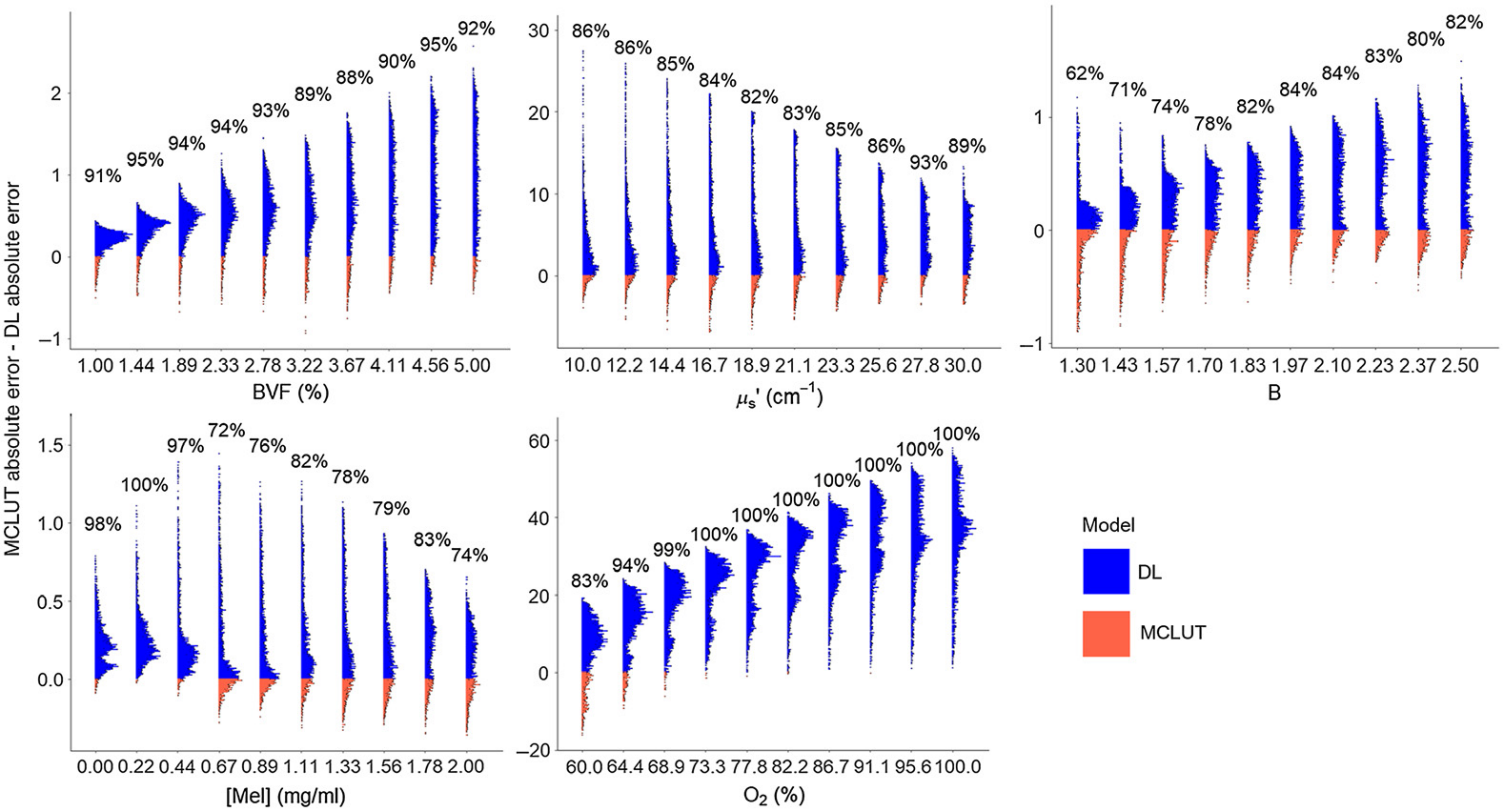
Distribution of MCLUT absolute error—DL absolute error for each parameter value. Percentages represent the rate at which DL is more accurate than MCLUT for that parameter. Blue represents where DL has lower error than MCLUT; orange represents where MCLUT has lower error than DL. While the DL model has the lower error most of the time for all parameter values, the model performance can vary for individual parameter values.

To analyze how the DL model performs compared to the MCLUT for individual parameter values, the difference in absolute error for each parameter is shown in Fig. 3. The percentages shown are the fractions of datapoints where this difference in error is positive, meaning DL had a lower error than MCLUT. For BVF and ${\mu_{s}}^{\prime }$, DL had lower errors most of the time, although it is still possible that MCLUT will perform better than DL for any one parameter value. The plot for B shows that for lower values of B, DL does not perform as well as the other values of B but still has the lower error overall. For Mel, DL is more accurate for Mel values less than 0.67  mg/ml, but still has lower error for the other values most of the time. Lastly, DL is very accurate for $O_{2}$, especially for $O_{2}$ values greater than 64.4% where DL almost always has the lower error.

The runtime to estimate parameters for each of the models used is shown in Table 2. When extracting parameters for one spectrum, the MCLUT computational time is on the same order as the H2O machine learning models. However, the MCLUT model has a trend of an increasing runtime as the number of spectra increases while the other H2O models do not share this trend. The H2O DL, RF, GBM, and GLM have similar runtimes of under 1 s across models and across the number of spectra, although there is some variance. The MATLAB DL model had notably faster computation times compared to all the machine learning models in H2O and the MCLUT model, with a reduction of at least two orders of magnitude. The MATLAB DL model was built for the purpose of a comparable time analysis. To ensure that this model still maintains the accuracy obtained from the machine learning model comparison, the mean percent errors were also calculated: 5.82% for BVF, 5.71% for ${\mu_{s}}^{\prime }$, 8.81% for B, 7.41% for Mel, and 4.74% for $O_{2}$. These percent errors are very similar to those from the H2O DL model. To further show the extent to which the two DL models from the different programming environments are comparable, refer to Fig. S3 in the Supplementary Material.

**Table 2:** Runtime comparison across trained models.

# spectra	MCLUT CPU time (s)	MATLAB DL CPU time (s)	H2O DL CPU time (s)	H2O RF CPU time (s)	H2O GBM CPU time (s)	H2O GLM CPU time (s)
1	0.67	0.00065	0.58	0.44	0.43	0.40
10	7.57	0.00084	0.45	0.40	0.39	0.41
50	38.92	0.0024	0.42	0.40	0.40	0.41
100	75.83	0.0033	0.41	0.50	0.60	0.45

## 4 Discussion

To extract physiological parameters from six wavelengths of diffuse reflectance spectra, several models were explored to determine the preferred model. The widely used MCLUT inverse model had the highest absolute errors compared to that of each of the machine learning models considered when only six wavelengths were used. The high errors are likely due to the inverse model not adjusting properly for the weighted contributions of the six-wavelength spectra. Out of the machine learning models used, GLM had the highest errors out of all the machine learning models for all parameters except $O_{2}$, which suggests that the data likely did not follow a normal distribution to fit a linear regression. The DL model had the lowest absolute errors across the parameters except for BVF and Mel, which have very close errors to the RF model. However, from the mixed model, which considers random effects from the true values of the parameters, DL was significantly different from each of the other models for all parameters. DL seems to be the most accurate overall because there are multiple layers that are able to describe the nonlinear relationship between spectra and parameters, and these layers are further refined in the learning process. The mean absolute percent errors for DL are all under 10%, which is comparable to what seems to be the acceptable error range of 1–10% found in literature for extracted optical properties.^13^^,^^22^^–^^26^ The absolute error difference plots show that the DL model performs better than the MCLUT model for all parameter values most of the time. Generally, the DL model works better for predicting BVF and $O_{2}$ while the predictions for B were weaker. Because the focus of this study was on the feasibility of using six wavelengths with machine learning and not on the translation to system measurements, the machine learning models used the default settings and were not optimized. Osman et al.^27^ studied the optimization of k-nearest neighbors and support vector machines and found that models vary in their sensitivity to hyperparameters that are adjusted for optimization and that optimized models can be as accurate or significantly more accurate. Therefore, before using any of the models in our study for actual measurements, the model should be optimized. The results from this study show that even with the default settings, a DL model can accurately predict parameters. Overall, we demonstrated that parameter extraction can be done accurately with six wavelengths using a DL model, and therefore devices with less wavelengths could be used to significantly lower the cost of the system.

In addition to lowering cost by limiting the number of wavelengths, the computational time to extract parameters will also be reduced independent of what model is used due to less data needed for collection and processing. We also compared the execution time of the trained models to determine if the machine learning models are advantageous over the MCLUT. While the MCLUT model is able to extract parameters for one spectrum at a similar speed to the H2O machine learning models, this runtime increases with the number of samples while the H2O machine learning models maintain the same speed. This is due to H2O’s in-memory distributed parallel processing that allows for multiple threads to compute at the same time, which is advantageous for large sample sizes. However, one would expect that machine learning models in general would be faster than the MCLUT model, which uses a nonlinear fitting algorithm. The MCLUT algorithm uses an iterative search, which is executed several times to fit the parameters, whereas machine learning models uses trained data to focus and speed up the search.^28^ This is then shown in the results when a similar DL model was built in MATLAB and the runtime is notably faster, which suggests that there is some amount of overhead time associated with communicating with the H2O cluster when using any of the H2O models. By building a DL model in MATLAB-based off the accuracy results from the H2O models, a fair comparison was able to be made against the MCLUT model for time analysis, which showed that DL had two or more orders of magnitude faster speeds for individual and multiple spectra. Being able to extract parameters for samples at a quicker speed could be meaningful for various situations such as patients with multiple lesions to be looked at, determining the exact boundaries of a lesion by collecting multiple spectra around the lesion, or possibly scanning larger sections of tissue for physiological parameter extraction. Typically, two to three spectra are collected for one lesion. If a patient has five suspicious lesions, 10 to 20 lesions are collected for the same patient. Then, if 10 patients’ data are processed at once, 100 to 200 lesions are used. The speed of machine learning models in this context would be useful for reducing the time for the evaluation of lesions.

For this study, the focus was on the computational processing to show the feasibility of using the 6-Channel device. The spectral responsivity of the 6-Channel device was taken from the datasheet for this purpose. For future experimental work with a DRS system, the device’s actual spectral responsivity will be characterized before taking measurements. Overall, DL is a promising machine learning tool to determine the physiological parameters from six wavelengths. Future work will include the optimization of the DL model and the assessment of the optimized model against the MCLUT model on phantoms with a DRS system that utilizes the 6-Channel sensor chip. From this, the accuracy of using DL with six wavelengths will be further evaluated with clinical data. DRS data has also been used for classification of various tissue lesions.^5^^,^^8^ Thus, after the accuracy is validated, parameter extraction can be used in combination with classification to allow clinicians to make a quantitatively informed diagnosis.

## 5 Conclusions

Machine learning, specifically DL models, are advantageous over the MCLUT inverse model for extracting physiological parameters from fewer wavelengths than a typical spectrometer uses in both speed and accuracy. By overcoming the limitations of the MCLUT model, the cost of a DRS system could be significantly decreased by using a chip such as the AS7262 6-Channel Visible Spectral ID device that only collects reflectance at six wavelengths. With reduced costs, a cheaper DRS system would enable more accessibility and use in the clinic to provide clinicians with information to differentiate tissue and aid the diagnosis of several applications.

## Code, Data, and Materials Availability

The code used to generate the results and figures is available in a Github repository: https://github.com/maynanguyen/Machine-Learning-for-Six-Wavelength-DRS

## References

[1] Zhu C. et al., “Diagnosis of breast cancer using fluorescence and diffuse reflectance spectroscopy: a Monte-Carlo-model-based approach,” J. Biomed. Opt. 13(3), 034015 (2008). JBOPFO 1083-3668 10.1117/1.2931078 18601560 PMC2791791

[2] Zonios G. et al., “Diffuse reflectance spectroscopy of human adenomatous colon polyps in vivo,” Appl. Opt. 38(31), 6628 (1999). APOPAI 0003-6935 10.1364/AO.38.006628 18324198

[3] Chang S. K. et al., “Combined reflectance and fluorescence spectroscopy for in vivo detection of cervical pre-cancer,” J. Biomed. Opt. 10(2), 024031 (2005). JBOPFO 1083-3668 10.1117/1.1899686 15910104

[4] Georgakoudi I. et al., “Trimodal spectroscopy for the detection and characterization of cervical precancers in vivo,” Am. J. Obstet. Gynecol. 186(3), 374–382 (2002). AJOGAH 0002-9378 10.1067/mob.2002.121075 11904594

[5] de Veld D. C. G. et al., “Autofluorescence and diffuse reflectance spectroscopy for oral oncology,” Lasers Surg. Med. 36(5), 356–364 (2005). LSMEDI 0196-8092 10.1002/lsm.20122 15856507

[6] Schwarz R. A. et al., “Autofluorescence and diffuse reflectance spectroscopy of oral epithelial tissue using a depth-sensitive fiber-optic probe,” Appl. Opt. 47(6), 825–834 (2008). APOPAI 0003-6935 10.1364/AO.47.000825 18288232 PMC2773166

[7] Spliethoff J. W. et al., “Improved identification of peripheral lung tumors by using diffuse reflectance and fluorescence spectroscopy,” Lung Cancer 80(2), 165–171 (2013). 10.1016/j.lungcan.2013.01.016 23402823

[8] Zhang Y. et al., “Physiological model using diffuse reflectance spectroscopy for nonmelanoma skin cancer diagnosis,” J. Biophotonics 12(12), e201900154 (2019). 10.1002/jbio.201900154 31325232

[r9] Rodriguez-Diaz E. et al., “Optical spectroscopy as a method for skin cancer risk assessment,” Photochem. Photobiol. 95(6), 1441–1445 (2019). PHCBAP 0031-8655 10.1111/php.13140 31287160

[10] Garcia-Uribe A. et al., “*In vivo* diagnosis of melanoma and nonmelanoma skin cancer using oblique incidence diffuse reflectance spectrometry,” Cancer Res. 72(11), 2738–2745 (2012). CNREA8 0008-5472 10.1158/0008-5472.CAN-11-4027 22491533 PMC3367032

[11] Vishwanath K. et al., “Portable, fiber-based, diffuse reflection spectroscopy (DRS) systems for estimating tissue optical properties,” Appl. Spectrosc. 65(2), 206–215 (2011). APSPA4 0003-7028 10.1366/10-06052 PMC3074566 21499501

[12] Rajaram N. Nguyen T. H. Tunnell J. W., “Lookup table–based inverse model for determining optical properties of turbid media,” J. Biomed. Opt. 13(5), 050501 (2008). JBOPFO 1083-3668 10.1117/1.2981797 19021373 PMC2627585

[13] Hennessy R. et al., “Monte Carlo lookup table-based inverse model for extracting optical properties from tissue-simulating phantoms using diffuse reflectance spectroscopy,” J. Biomed. Opt. 18(3), 037003 (2013). JBOPFO 1083-3668 10.1117/1.JBO.18.3.037003 23455965 PMC3584151

[14] Zhong X. Wen X. Zhu D., “Lookup-table-based inverse model for human skin reflectance spectroscopy: two-layered Monte Carlo simulations and experiments,” Opt. Express 22(2), 1852–1864 (2014). OPEXFF 1094-4087 10.1364/OE.22.001852 24515194

[15] Barman I. et al., “Rapid and accurate determination of tissue optical properties using least-squares support vector machines,” Biomed. Opt. Express 2(3), 592–599 (2011). BOEICL 2156-7085 10.1364/BOE.2.000592 21412464 PMC3047364

[16] Tsui S.-Y. et al., “Modelling spatially-resolved diffuse reflectance spectra of a multi-layered skin model by artificial neural networks trained with Monte Carlo simulations,” Biomed. Opt. Express 9(4), 1531–1544 (2018). BOEICL 2156-7085 10.1364/BOE.9.001531 29675300 PMC5905904

[17] Panigrahi S. Gioux S., “Machine learning approach for rapid and accurate estimation of optical properties using spatial frequency domain imaging,” J. Biomed. Opt. 24(7), 071606 (2018). JBOPFO 1083-3668 10.1117/1.JBO.24.7.071606 PMC6995874 30550050

[18] Kejela G. Esteves R. M. Rong C., “Predictive analytics of sensor data using distributed machine learning techniques,” in Proc. Int. Conf. Cloud Comput. Technol. and Sci., CloudCom 2015-February, IEEE Computer Society, pp. 626–631, (2015).

[19] Candel A. et al., Deep Learning with H2O, 5th ed., H2O.ai, Mountain View, California (2016).

[20] “Algorithms—H2O 3.30.0.3 documentation,” http://docs.h2o.ai/h2o/latest-stable/h2o-docs/data-science.html (accessed 15 May 2020).

[21] Singmann H. et al., “afex: analysis of factorial experiments. R package version 0.20-2” (2018).

[22] Bish S. F., “Development of a noncontact diffuse optical spectroscopy probe for measuring tissue optical properties,” J. Biomed. Opt. 16(12), 120505 (2011). JBOPFO 1083-3668 10.1117/1.3662459 22191909 PMC3247933

[r23] Bender J. E. et al., “A robust Monte Carlo model for the extraction of biological absorption and scattering in vivo,” IEEE Trans. Biomed. Eng. 56(4), 960–968 (2009). IEBEAX 0018-9294 10.1109/TBME.2008.2005994 19423425 PMC2791541

[r24] Yu B. et al., “Diffuse reflectance spectroscopy of epithelial tissue with a smart fiber-optic probe,” Biomed. Opt. Express 5(3), 675–689 (2014). BOEICL 2156-7085 10.1364/BOE.5.000675 24688805 PMC3959852

[r25] Edwards P. et al., “Smartphone based optical spectrometer for diffusive reflectance spectroscopic measurement of hemoglobin,” Sci. Rep. 7, 1 (2017). SRCEC3 2045-2322 10.1038/s41598-016-0028-x 28939898 PMC5610341

[26] Gunther J. Lu H. Andersson-Engels S., “Combination of diffuse reflectance and transmittance spectroscopy to obtain optical properties of liquid phantoms,” Opt. Eng. 59(2), 024109 (2020). 10.1117/1.OE.59.2.024109

[27] Osman H. Ghafari M. Nierstrasz O., “Hyperparameter optimization to improve bug prediction accuracy,” in MaLTeSQuE 2017—IEEE Int. Workshop on Mach. Learn. Tech. for Software Quality Eval., co-located with SANER 2017, Institute of Electrical and Electronics Engineers Inc., pp. 33–38 (2017).

[28] Agakov F. et al., “Using machine learning to focus iterative optimization,” in Proc. CGO 2006—The 4th Int. Symp. Code Gener. and Optim., pp. 295–305 (2006).

