# Supplementary material for: Machine learning to extract physiological parameters from multispectral diffuse reflectance spectroscopy
Source: J Biomed Opt. 2021 Mar 17;26(5):052912. doi: 10.1117/1.JBO.26.5.052912 (PMC7969972; doi:10.1117/1.JBO.26.5.052912)
Supplement: Supplementary file 1 [file JBO_026_052912_SD001.pdf]

Supplementary Material:
Machine learning to extract physiological parameters from 6-wavelength diffuse reflectance spectroscopy

Mayna H. Nguyen,[a] Yao Zhang,[a] Frank Wang,[a] Jose De La Garza Evia,[a] Mia K. Markey,[a,b] James W. Tunnell[a,*]
[a]University of Texas at Austin, Department of Biomedical Engineering, 107 W. Dean Keeton Street, Austin, Texas 78712, United States
[b]University of Texas MD Anderson Cancer Center, 1515 Holcombe Boulevard, Houston, Texas 77030, United States

Spectral Responsivity Figure

Fig. S1 shows the spectral responsivity from the AS7262 6-Channel Visible Spectral ID Device datasheet provided by ams. A weighted average approach was used to calculate the reflectance values at the 6 wavelengths according to the normal distributions shown in the device specifications.

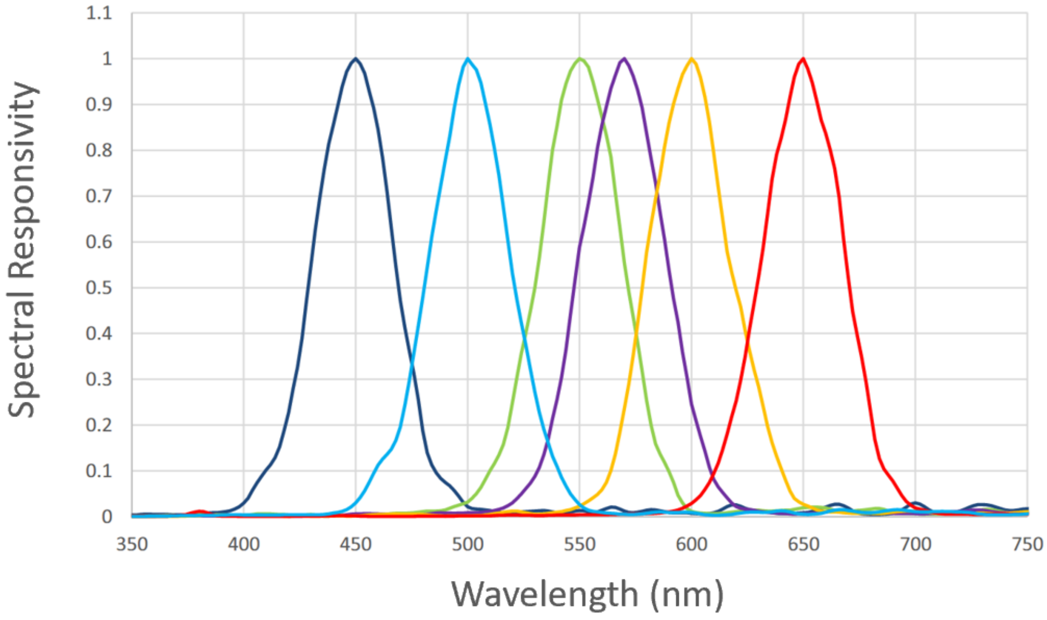

Fig. S1 Spectral responsivity shows normal distributions at each of the wavelengths of the AS7262 6-Channel Visible Spectral ID Device: 450 nm, 500 nm, 550 nm, 570 nm, 600 nm, and 650 nm.

Noise Results and Analysis

The mean absolute percent errors of the MCLUT and H2O DL model with Gaussian, Poisson, and pink noise are shown in Table S1. From these errors, it is clear that the addition of noise does not have a major effect on the results for both DL and MCLUT models. The MCLUT has much larger errors in general compared to the DL model for with and without noise, demonstrating the robustness of the DL method.

Table S1 Mean absolute percent errors of MCLUT and H2O DL models with various noise types

	**MCLUT no noise**	**MCLUT Gaussian noise**	**MCLUT Poisson noise**	**MCLUT Pink noise**	**H2O DL no noise**	**H2O DL Gaussian noise**	**H2O DL Poisson noise**	**H2O DL Pink noise**
**BVF**	25.25%	25.26%	26.29%	25.36%	5.86%	5.15%	5.50%	6.37%
**μ_s'**	28.98%	29.11%	29.16%	29.05%	6.69%	6.88%	7.14%	7.55%
**B**	22.89%	22.93%	22.25%	23.12%	9.54%	9.81%	9.82%	10.33%
**Mel**	39.76%	39.94%	39.64%	39.89%	9.80%	9.91%	9.72%	9.66%
**O_2**	35.41%	35.29%	34.82%	35.12%	4.37%	4.61%	5.23%	6.09%

Absolute Percent Error Figure

The absolute percent error distribution is shown in Fig. S2. Due to division by small parameter values, there is high variance in both models, despite the mean absolute percent error of DL being less than 10% for all parameters. Although there is variance within DL errors, most of the error is centered around 0. MCLUT has exceptionally high variance for μ_s' and Mel parameters.

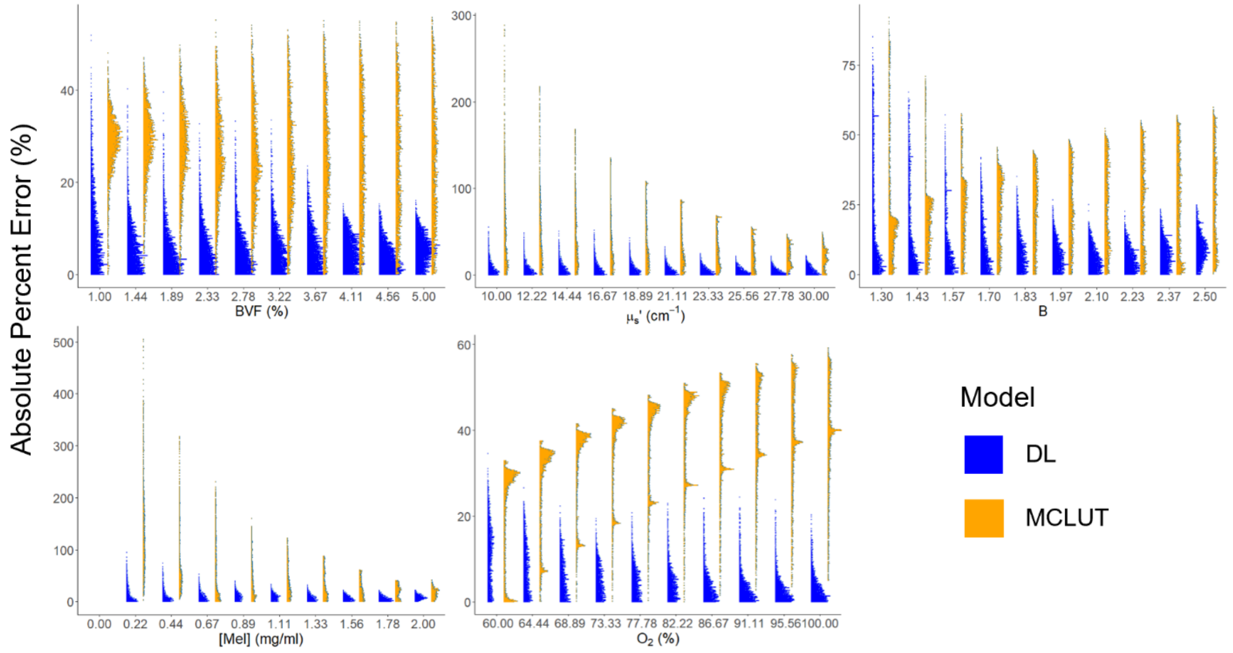

Fig. S2 Distributions of absolute percent error (%) using MCLUT (orange) and DL (blue) models for physiological parameter values. Mel distribution at 0 not shown because percent values are invalid due to division by zero. Despite DL having low mean percent errors, both MCLUT and DL have high variance for absolute percent error due to division by small parameter values.

MATLAB DL vs H2O DL Figure

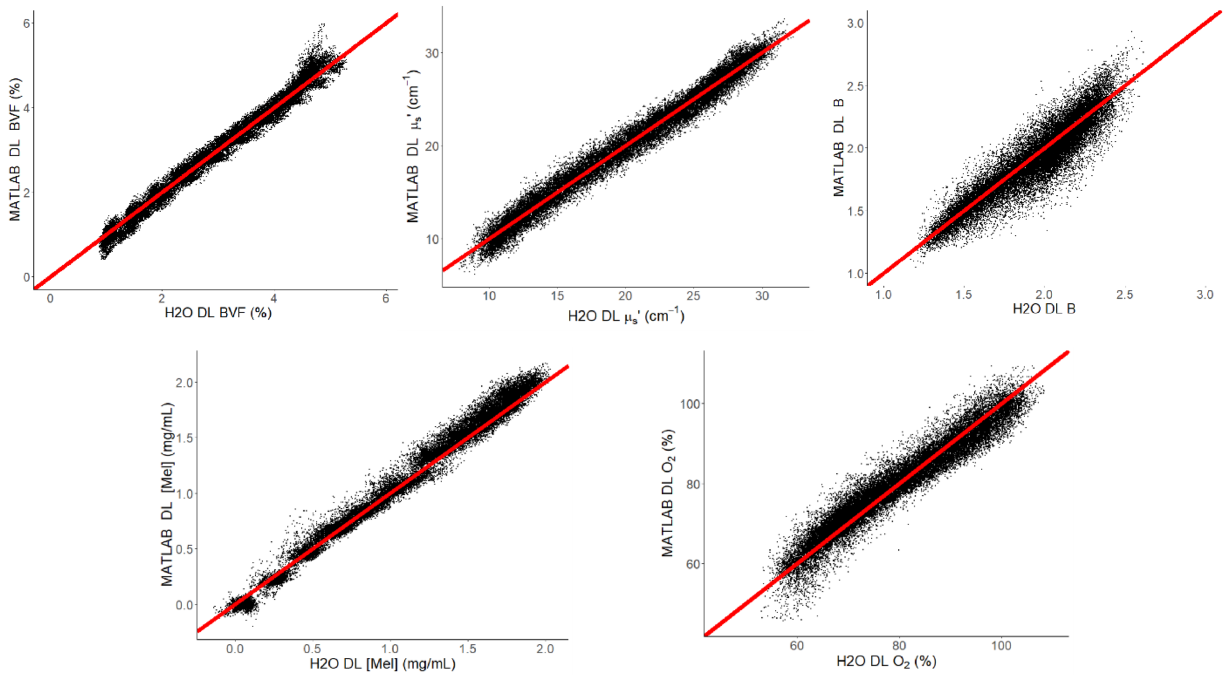

Fig. S3 Plots of MATLAB DL vs H2O DL for each of the five physiological parameters (BVF, μ_s', B, Mel, O_2). Identity line is shown in red to show how close the MATLAB and H2O deep learning models were in predicting parameter values.
